# Iron and Phosphate Deficiency Regulators Concertedly Control Coumarin Profiles in *Arabidopsis thaliana* Roots During Iron, Phosphate, and Combined Deficiencies

**DOI:** 10.3389/fpls.2019.00113

**Published:** 2019-02-11

**Authors:** Ranju Chutia, Steffen Abel, Jörg Ziegler

**Affiliations:** Department of Molecular Signal Processing, Leibniz Institute of Plant Biochemistry, Halle, Germany

**Keywords:** *Arabidopsis thaliana*, phosphate deficiency, iron deficiency, metabolite profiling, coumarins, regulation

## Abstract

Plants face varying nutrient conditions, to which they have to adapt to. Adaptive responses are nutrient-specific and strategies to ensure supply and homeostasis for one nutrient might be opposite to another one, as shown for phosphate (P_i_) and iron (Fe) deficiency responses, where many genes are regulated in an opposing manner. This was also observed on the metabolite levels. Whereas root and exudate levels of catechol-type coumarins, phenylpropanoid-derived 2-benzopyranones, which facilitate Fe acquisition, are elevated after Fe deficiency, they are decreased after P_i_ deficiency. Exposing plants to combined P_i_ and Fe deficiency showed that the generation of coumarin profiles in *Arabidopsis thaliana* roots by P_i_ deficiency considerably depends on the availability of Fe. Similarly, the effect of Fe deficiency on coumarin profiles is different at low compared to high P_i_ availability. These findings suggest a fine-tuning of coumarin profiles, which depends on Fe and P_i_ availability. T-DNA insertion lines exhibiting aberrant expression of genes involved in the regulation of P_i_ starvation responses (*PHO1, PHR1, bHLH32, PHL1, SPX1*) and Fe starvation responses (*BRUTUS, PYE, bHLH104, FIT*) were used to analyze the regulation of the generation of coumarin profiles in *Arabidopsis thaliana* roots by P_i_, Fe, and combined P_i_ and Fe deficiency. The analysis revealed a role of several Fe-deficiency response regulators in the regulation of Fe and of P_i_ deficiency-induced coumarin profiles as well as for P_i_ deficiency response regulators in the regulation of P_i_ and of Fe deficiency-induced coumarin profiles. Additionally, the regulation of Fe deficiency-induced coumarin profiles by Fe deficiency response regulators is influenced by P_i_ availability. Conversely, regulation of P_i_ deficiency-induced coumarin profiles by P_i_ deficiency response regulators is modified by Fe availability.

## Introduction

Coumarins are a group of compounds derived from the phenylpropanoid pathway. They possess a 2-benzopyranone core structure and individual members of this class of compounds exhibit different substitution patterns (Shimizu, 2014). Scopoletin, the first coumarin-specific intermediate in coumarin biosynthesis, contains a methoxy group and a hydroxyl group at positions 6 and 7, respectively (Supplementary Figure S1). Other coumarins contain a catechol moiety, which can either be demethylated scopoletin (esculetin), or mono- and dihydroxlated scopoletin (fraxetin and sideritin) (Kai et al., 2008; Shimizu, 2014; Sisó-Terraza et al., 2016; Rajniak et al., 2018; Siwinska et al., 2018; Tsai et al., 2018). In the model plant *Arabidopsis thaliana*, these coumarins have predominantly been detected in root exudates, whereas their respective monoglucosides, such as scopolin, esculin, fraxin, and sideritin glucoside, are almost exclusively found in root extracts (Fourcroy et al., 2014, 2016; Schmid et al., 2014; Schmidt et al., 2014; Sisó-Terraza et al., 2016; Ziegler et al., 2017; Rajniak et al., 2018; Siwinska et al., 2018; Tsai et al., 2018).

In recent years, it was shown that biosynthesis and exudation of coumarins, especially of catechol type coumarins, contribute to iron acquisition under iron limiting conditions (Rodríguez-Celma et al., 2013; Fourcroy et al., 2014, 2016; Schmid et al., 2014; Schmidt et al., 2014; Sisó-Terraza et al., 2016; Rajniak et al., 2018; Siwinska et al., 2018; Tsai et al., 2018). It was shown that iron deficiency-induced accumulation of coumarins was under positive control of the major regulator of iron deficiency responses FIT (FER-like Iron deficiency-induced Transcription Factor) (Schmid et al., 2014), a basic helix loop helix type transcription factor (bHLH 29) (Colangelo and Guerinot, 2004). FIT is part of a comprehensive network of transcriptional regulators required to orchestrate responses which enable the plant to cope with iron limiting conditions. Although many responses seem to be regulated by FIT alone, its interaction with the bHLH type transcription factors 38, 39, 100, and 101 is required to mediate a subset of iron deficiency responses (Colangelo and Guerinot, 2004; Yuan et al., 2008; Wang et al., 2013). Expression of *bHLH38*, *39*, *100*, and *101* is positively regulated by the transcription factor bHLH 104 (Li et al., 2016), which was shown to interact with BRUTUS (BTS), an E3 ubiquitin ligase protein with metal ion binding and DNA binding domains (Selote et al., 2015; Zhang et al., 2015). BTS, a negative regulator, is assumed to be responsible for fine tuning of iron deficiency responses by monitoring iron status through its metal ion binding domain (Kobayashi et al., 2013; Selote et al., 2015). Opposing effects on plant growth and development also suggest an interaction of BTS with the bHLH transcription factor POPEYE (PYE), which positively regulates several iron deficiency responses (Long et al., 2010). However, with the exception of FIT, the contribution of these regulators to iron deficiency-induced coumarin accumulation has not been investigated so far.

Recently, we observed changes in the levels of coumarins in Arabidopsis root exudates as well as of coumarin glucoside in Arabidopsis roots after plants have been subjected to phosphate (P_i_) deficiency (Ziegler et al., 2016). We observed an accumulation for a subset of coumarins, i.e., esculin, esculetin, scopolin, and scopoletin, whereas the levels of sideritin and its glucoside were strongly decreased, thus contrasting the iron deficiency-induced accumulation of all coumarins, especially of highly oxygenated ones, such as sideritin (Schmid et al., 2014; Rajniak et al., 2018). Several regulators of P_i_ deficiency responses have been described. The major regulator is PHR1, which binds to the PBS domain in the promoter of many phosphate starvation genes, thereby inducing their transcription (Rubio et al., 2001; Bustos et al., 2010; Briat et al., 2015). SPX proteins, for which three isoforms are known, negatively regulate P_i_ deficiency-induced gene expression. High intracellular P_i_ concentrations promote the interaction between PHR1 and SPX preventing PHR1 to bind to its target sites on the promoters. At low intracellular P_i_ concentrations, PHR1 is released from the PHR1-SPX complex, allowing it to activate gene expression (Puga et al., 2014; Wang et al., 2014). PHR1 was shown to regulate the transcription of about 60% of P_i_ starvation responsive genes (Bustos et al., 2010). Other genes, which are not controlled by PHR1, are mainly regulated by PHL transcription factors (PHR1-Like), for which several isoforms have been described (Bustos et al., 2010). Although conceivable, it has not yet been elucidated whether or not regulation by PHL proceeds through P_i_ mediated interaction with SPX protein, as is the case with PHR1. On the other hand, negative regulation of P_i_ starvation responses by the transcription factor bHLH32 was shown to be independent of P_i_ content (Chen et al., 2007). PHOSPHATE1 (PHO1) was identified as eukaryotic P_i_ exporter. Mutants with impaired *PHO 1* expression showed reduced shoot but increased root P_i_ levels indicating a disturbed shoot to root distribution of P_i_ (Poirier et al., 1991; Hamburger et al., 2002). Reduced transcriptional activation of P_i_ starvation response genes despite low P_i_ levels in shoots of *pho1* mutants as well as the presence of known P_i_ regulatory motifs in the PHO1 protein suggests a role for PHO1 in the coordination of P_i_ starvation responses (Rouached et al., 2011; Wege et al., 2016). Although many P_i_ starvation responses are controlled by these regulators, their involvement in the changes in P_i_ deficiency-induced coumarin profiles is unknown.

The different coumarin profiles observed after either P_i_ or Fe deficiency treatments alone, especially the opposing effects on sideritin levels (Ziegler et al., 2016), raises the question on the response after combined deficiency, specifically, whether withdrawal of Fe affects P_i_ deficiency induced changes in coumarin levels and vice versa. Furthermore, as mentioned above, FIT has been shown to play an important role in the accumulation of coumarins after Fe deficiency (Schmid et al., 2014), but no regulator of P_i_ deficiency induced alteration in coumarin profiles have been identified yet. Also, regulators of possible modifications of Fe deficiency-induced coumarin profiles by P_i_ deficiency and vice versa are not known.

In this report, we studied the effect of P_i_ deficiency, Fe deficiency, and combined P_i_ and Fe deficiency on coumarin profiles in *Arabidopsis thaliana* roots. Several mutants were included in order to elucidate the contribution of either P_i_ or Fe deficiency response regulators in the establishment of coumarin profiles. Since we were mainly interested in the regulation of coumarin biosynthesis rather than on coumarin exudation, we focused our analysis on coumarin glucosides, which are almost exclusively found inside roots and which most likely represent the major storage form of newly biosynthesized coumarins before being cleaved and exuded. Additionally, we could recently show that coumarin glucoside profiles obtained from root tissue are qualitatively and quantitatively correlated to coumarin aglycone profiles in exudates (Ziegler et al., 2016, 2017).

## Materials and Methods

### Plant Lines and Growth Conditions

*Arabidopsis thaliana* accession Columbia (Col-0) was used as WT throughout the study. All T-DNA insertion lines (Col-0 background) were provided by the Nottingham Arabidopsis Stock Center (NASC). Homozygous plants were generated by selfing heterozygous plants, and homozygosity was confirmed by PCR with the primers listed in Supplementary Table S1.

Seeds were surface sterilized with chlorine gas and individually placed with a toothpick on sterile agar plates containing 5 mM KNO_3_, 0.5 mM KH_2_PO_4_, 2 mM MgSO_4_, 2 mM Ca(NO_3_)_2_, 50 μM Fe-EDTA, 70 μM H_3_BO_3_, 14 μM MnCl_2_, 0.5 μM CuSO_4_, 1 μM ZnSO_4_, 0.2 μM Na_2_MoO_4_, 10 μM CoCl_2_, and 5 g l^-1^ of sucrose buffered with 2.5 mM Mes-KOH to pH 5.6. For -P_i_ medium, the concentration of KH_2_PO_4_ was reduced to 5 μM, for -Fe medium, Fe-EDTA was omitted. Agar (Phyto Agar, Duchefa, Haarlem, Netherlands) was routinely purified as described (Ward et al., 2008) and added to a concentration of 1% (w/v). Plates were incubated for 2 days in the dark at 4°C to synchronize seed germination. Afterward, agar plates were kept in a vertical position in a growth chamber at 22°C under illumination for 16 h daily (170 μmol s^-1^ m^-2^; Osram LumiluxDeLuxe Cool daylight L58W/965, Osram, Augsburg, Germany). After 5 days of growth, plants were transferred to fresh agar plates containing the respective conditions (+P_i_,+Fe; -P_i_,+Fe; +P_i_,-Fe; -P_i_,-Fe). After additional 6 days of growth, roots were separated from the shoots, their fresh weight recorded and frozen in liquid nitrogen until further processing. One biological replicate consisted of roots from two plants (coumarin concentration) or shoots and roots from one plant (P_i_ concentration). The phenotypes of WT plants and mutant plants grown under the four applied treatments were recorded at the time of harvest after and are shown in Supplementary Figure S2.

### Metabolite Analysis

Frozen tissues (1–5 mg of fresh weight) were ground using 5 mm steel beads in a bead mill at 25 s^-1^ for 50 s, and the resulting powder was extracted by vigorous shaking for 20 min with 100 μl of 70% (v/v) methanol containing 2 nmol of 4-methyl-umbelliferon and 5 nmol [2,2,3,3-^2^H] succinic acid as internal standards for coumarin and P_i_ quantification, respectively. Targeted coumarin profiling was performed as described (Ziegler et al., 2016, 2017). For the determination of P_i_ concentrations, 10 μl of the extracts were evaporated to dryness, methoxylated with 20 μl of 20 mg ml^-1^ of methoxyamine in pyridine (Sigma-Aldrich, St. Louis, MO, United States) for 1.5 h at room temperature, and silylated for 30 min at 37°C with 35 μl of Silyl 991 (Macherey-Nagel, Düren, Germany). Gas chromatography (GC)-MS/MS was performed as described (Ziegler et al., 2016) with some modifications. Briefly, the Agilent 7890 GC system was equipped with an OPTIMA 5 column (10 m × 0.25 mm, 0.25 μm; Macherey-Nagel, Düren, Germany) and coupled to an Agilent 7000B triple quadrupole mass spectrometer operated in the positive chemical ionization mode (reagent gas: methane, gas flow: 20%, ion source temperature; 230°C). One microliter was injected [pulsed (25 psi) splitless injection] at 220°C. The initial temperature of 60°C was held for 1 min, followed by increases at 35°C min^-1^ to 200°C and 50°C min^-1^ to 340°C. The final temperature of 340°C was held for 5 min. Helium was used as the carrier at 2.39 ml min^-1^. The transfer line was set to a temperature of 250°C. Helium and N_2_ were used as quench and collision gasses, respectively (2.25 and 1.5 ml min^-1^). Multiple reaction monitoring parameters for the detection of P_i_ (3TMS) and [2,2,3,3-^2^H] succinic acid (2TMS) are indicated in Supplementary Table S2. The IntelliQuant algorithm of the Analyst 1.6.2 software (AB Sciex, Darmstadt, Germany) or the Agile algorithm of the MassHunter Quantitative Analysis software (version B06.00, Agilent, Waldbronn, Germany) were used to integrate the peaks for coumarins or P_i_, respectively. Coumarins and P_i_ concentrations were quantified using 4-methyl-umbelliferon and [2,2,3,3-^2^H] succinic acid, respectively, and the calculated amounts were divided by the fresh weight. In order to account for variations in absolute coumarin concentrations between independent experiments, all values within individual experiments were normalized to the average values of the biological replicates of the Col0 +P_i_+Fe treatment in the respective experiment. The raw data are available in Supplementary Dataset S1.

## Results

### Root Coumarin Profiles in Response to P_i_ Deficiency, Fe Deficiency, and Combined P_i_ and Fe Deficiency

**FIGURE 1:**
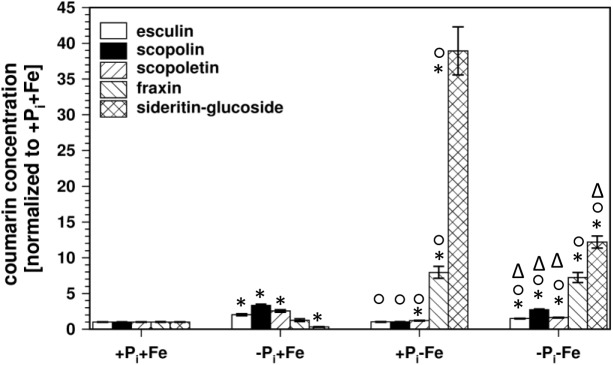
Normalized coumarin concentrations in roots of WT plants (normalized to +P_i_+Fe, average absolute amounts: esculin (open bars): 0.64 pmol mg^-1^ FW; scopolin (solid bars): 702 pmol mg^-1^ FW; scopoletin (hatched/bars): 16.8 pmol mg^-1^ FW; fraxin (hatched∖bars): 1.9 pmol mg^-1^ FW; sideritin glucoside (cross hatched bars): 3.6 pmol mg^-1^ FW). Plants were grown for 5 days on +P_i_+Fe agar plates, transferred to the indicated conditions and allowed to grow for additional 6 days before harvest. +P_i_: 500 μM, –P_i_: 5 μM, +Fe: 50 μM, –Fe: no Fe added. Error bars indicate SE (*n* ≥ 35). Significance analyses between treatments were performed by Student’s *t*-test (two tailed, equal variances). ^∗^*P* ≤ 0.05 compared to +P_i_+Fe; °*P* ≤ 0.05 compared to –P_i_+Fe; ^Δ^*P* ≤ 0.05 compared to +P_i_–Fe.

At first we analyzed the coumarin glucoside profile in roots of Col0 wild-type plants after P_i_ and Fe deficiency, as well as after combined deficiency (Figure 1). In P_i_ depleted conditions (-P_i_, +Fe), sideritin glucoside concentration in roots was decreased by about 70%, whereas esculin, scopolin, and scopoletin levels increased two- to three-fold. The slight increase in the concentration of fraxin (1.2-fold) was of low statistical significance (*P* = 0.22). As reported (Schmid et al., 2014; Schmidt et al., 2014; Sisó-Terraza et al., 2016; Rajniak et al., 2018; Tsai et al., 2018), fraxin and sideritin glucoside levels strongly increased almost 8- and 40-fold, respectively, after Fe deficiency (+P_i_, -Fe). For esculin and scopolin, statistically significant (*P* ≤ 0.05) changes could not be detected, whereas scopoletin levels slightly increased by 20%. Interestingly, Fe deficiency-induced sideritin glucoside levels were lower in case plants experienced additional P_i_ limitation (-P_i_, -Fe). However, the amount of fraxin was not affected. After combined deficiency, esculin, scopolin, and scopoletin levels approached those observed after P_i_ deficiency alone (-Pi, +Fe), but were still significantly (*P* ≤ 0.05) lower. Consistently, the P_i_ deficiency response, decrease in sideritin glucoside and accumulation of esculin, scopolin, and scopoletin, was different in the absence than in the presence of Fe (two way ANOVA: *P* ≤ 0.01). Similarly, the Fe deficiency response was dependent on the applied P_i_ concentration. Whereas the increase in sideritin glucoside levels was lower in the absence compared to the presence of P_i_ (two way ANOVA: 5.8 e^-12^), fraxin accumulation was unaffected, and esculin, scopolin, and scopoletin levels rather declined (two way ANOVAs: *P* = 6.5 e^-4^, 6.9 e^-3^, 1.6 e^-7^, respectively). These results indicate a cross-talk between Fe and P_i_ nutrition in the generation of coumarin profiles, especially the impact of P_i_ limitation on Fe deficiency-induced coumarin accumulation. In order to evaluate whether this is due to internal or external P_i_ concentrations, we measured the P_i_ content in roots and shoots.

### Correlation Between Coumarin Profiles and P_i_ Content

**FIGURE 2:**
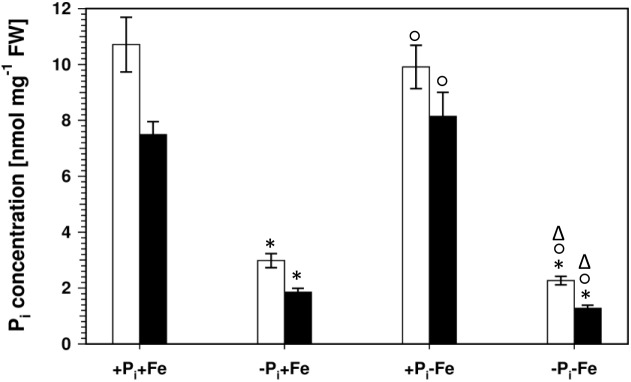
P_i_ concentrations in roots (open bars) and shoots (solid bars) of WT plants. Plants were grown for 5 days on +P_i_+Fe agar plates, transferred to the indicated conditions and allowed to grow for additional 6 days before harvest. +P_i_ : 500 μM, –P_i_ : 5 μM, +Fe: 50 μM, –Fe: no Fe added. Error bars indicate SE (*n* ≥ 35). Significance analyses between treatments were performed by Student’s *t*-test (two tailed, equal variances). ^∗^*P* ≤ 0.05 compared to +P_i_+Fe; °*P* ≤ 0.05 compared to –P_i_+Fe; ^Δ^*P* ≤ 0.05 compared to +P_i_–Fe.

**FIGURE 3:**
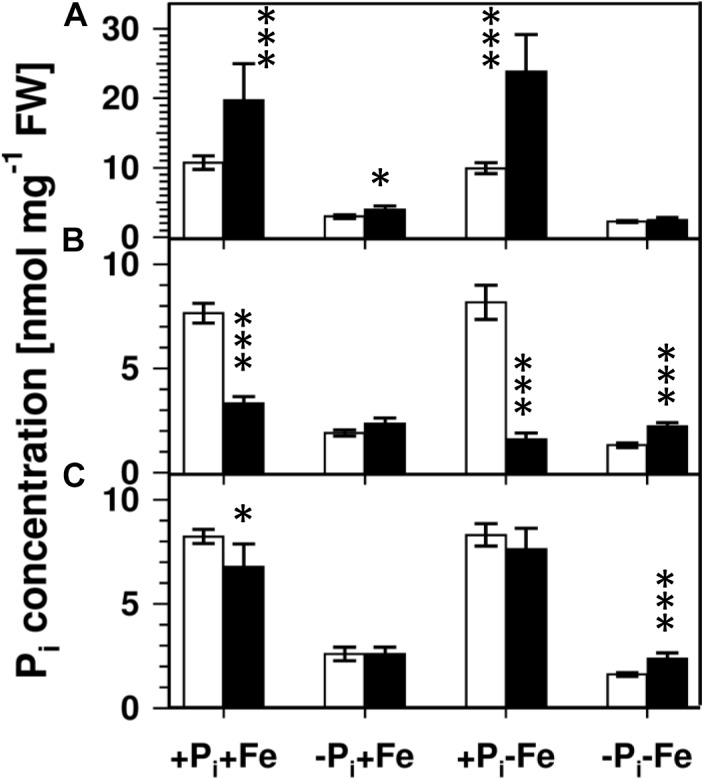
P_i_ concentrations in roots **(A)**, shoots **(B)**, and seedlings **(C)** of WT (open bars) and *pho1* (solid bars). Plants were grown for 5 days on +P_i_+Fe agar plates, transferred to the indicated conditions and allowed to grow for additional 6 days before harvest. +P_i_ : 500 μM, –P_i_ : 5 μM, +Fe: 50 μM, –Fe: no Fe added. Error bars indicate SE (*n* ≥ 8). Significance analyses between *pho1* and Col0 were performed by Student’s *t*-test (two tailed, equal variances): ^∗^*P* ≤ 0.05; ^∗∗^*P* ≤ 0.01; ^∗∗∗^*P* ≤ 0.001.

**FIGURE 4:**
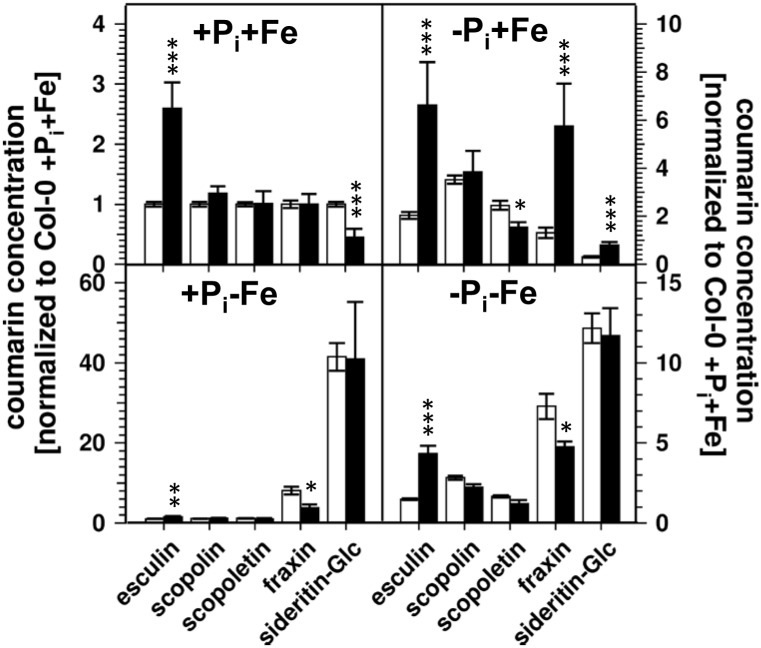
Normalized coumarin concentrations in roots of WT (open bars) and *pho1* (solid bars) plants (normalized to Col-0 +P_i_+Fe, average absolute amounts: esculin: 0.64 pmol mg^-1^ FW; scopolin: 702 pmol mg^-1^ FW; scopoletin: 16.8 pmol mg^-1^ FW; fraxin: 1.9 pmol mg^-1^ FW; sideritin glucoside: 3.6 pmol mg^-1^ FW). Plants were grown for 5 days on +P_i_ +Fe agar plates, transferred to the indicated conditions and allowed to grow for additional 6 days before harvest. +P_i_: 500 μM, –P_i_: 5 μM, +Fe: 50 μM, –Fe: no Fe added. Error bars indicate SE (*n* ≥ 8). Significance analyses between *pho1* and Col0 were performed by Student’s *t*-test (two tailed, equal variances): ^∗^*P* ≤ 0.05; ^∗∗^*P* ≤ 0.01; ^∗∗∗^*P* ≤ 0.001.

Lowering the P_i_ concentration in the medium from 500 to 5 μM resulted in a decrease of root P_i_ by 70% (Figure 2). Simultaneous Fe deficiency (-P_i_, -Fe) led to a further decrease by 30%, whereas growth in Fe-depleted conditions alone (+P_i_, -Fe) did not alter P_i_ levels. Shoot P_i_ levels followed a similar pattern. From these data it seems that decreased P_i_ levels lead to decreased sideritin glucoside, and increased esculin, scopolin, and scopoletin concentrations irrespective of the presence of iron, suggesting that coumarin profiles in roots of plants grown in control as well as in Fe deficient conditions are modulated by the internal P_i_ status. To further elaborate such a correlation we analyzed the *pho1* mutant which exhibits an aberrant P_i_ distribution between roots and shoots (Figure 3) (Poirier et al., 1991; Hamburger et al., 2002; Rouached et al., 2011). In case changes in sideritin glucoside, esculin, scopolin, and scopoletin levels would be due to root P_i_ status, roots of *pho1* plants showing higher P_i_ concentrations should exhibit increased sideritin glucoside and decreased esculin, scopolin, and scopoletin levels compared to WT. However, by comparing Figure 3, 4, *pho1* roots rather displayed lower sideritin glucoside and increased esculin levels compared to WT under nutrient sufficient conditions (+P_i_,+Fe) despite higher P_i_ concentrations in roots. Also, sideritin glucoside and esculin levels in *pho1* roots exposed to Fe deficiency (+P_i_, -Fe) are similar or slightly increased, respectively, although root P_i_ levels are higher. Only the elevated P_i_ levels in *pho1* roots exposed to P_i_ limiting conditions (-P_i_, +Fe) coincided with higher sideritin glucoside levels, but esculin and fraxin concentrations were strongly elevated compared to WT. Considering the P_i_ status of whole seedlings grown in nutrient sufficient condition (+P_i_, +Fe), lower levels of P_i_ in *pho1* corresponded to decreased sideritin glucoside and increased esculin levels in *pho*1 roots compared to WT, suggesting that the levels of sideritin glucoside and esculin levels are negatively and positively correlated, respectively, to the P_i_ concentration of the whole seedling. However, although seedling P_i_ levels were higher in *pho1* compared to WT under conditions of combined deficiency (-P_i_, -Fe), sideritin glucoside levels were indistinguishable between the mutant and Col0, and esculin levels were about threefold higher in *pho1.* These results show that the effect of P_i_ deficiency on coumarin profiles can only be partially attributed to cellular P_i_ concentration. Remarkably, P_i_ limitation resulted in a strong accumulation of fraxin in roots of P_i_ starved *pho1* roots, which was not observed in WT roots.

The observation that coumarin profiles are modified by P_i_ starvation raised the question whether P_i_ deficiency response or Fe deficiency response regulators are involved. Therefore, T-DNA lines harboring insertions in several known regulatory genes of both responses were tested for their coumarin profiles under the four conditions described above.

### Effect of Fe Deficiency Response Regulator Mutants on Coumarin Profiles

**FIGURE 5:**
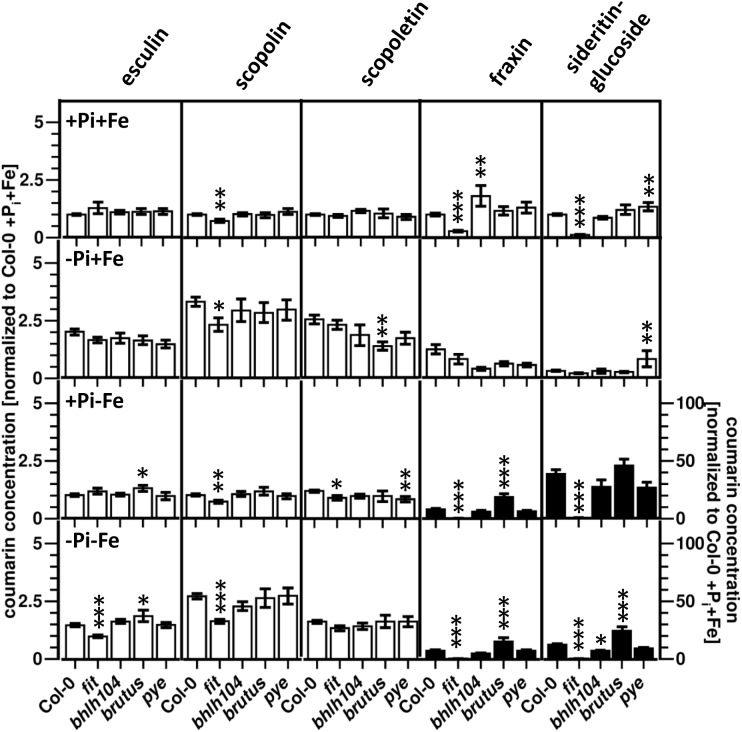
Normalized coumarin concentrations in roots of WT, *fit*, *bhlh104*, *brutus*, and *pye* plants (normalized to Col-0 +P_i_+Fe, average absolute amounts: esculin: 0.64 pmol mg^-1^ FW; scopolin: 702 pmol mg^-1^ FW; scopoletin: 16.8 pmol mg^-1^ FW; fraxin: 1.9 pmol mg^-1^ FW; sideritin glucoside: 3.6 pmol mg^-1^ FW). Plants were grown for 5 days on +P_i_+Fe agar plates, transferred to the indicated conditions and allowed to grow for additional 6 days before harvest. +P_i_: 500 μM, –P_i_: 5 μM, +Fe: 50 μM, –Fe: no Fe added. Error bars indicate SE (*n* ≥ 8). Significance analyses between mutants and Col0 were performed by Student’s *t*-test (two tailed, equal variances): ^∗^*P* ≤ 0.05; ^∗∗^*P* ≤ 0.01; ^∗∗∗^*P* ≤ 0.001. Note that the values for fraxin and sideritin glucoside in the lower two panels (black bars) refer to the y-axis to the right.

The *fit* mutant, which is deficient in the expression of a regulator mediating many Fe deficiency responses, was previously shown to exhibit reduced root fluorescence after Fe deficiency compared to WT, indicating lower coumarin accumulation (Schmid et al., 2014). Our analysis showed that sideritin glucoside and fraxin levels were strongly reduced (50- and 20- fold, respectively) in these plants compared to WT after exposure to Fe limiting conditions (+P_i_, -Fe), scopolin and scopoletin levels were diminished by 30%, whereas esculin levels were indistinguishable to WT (Figure 5 and Supplementary Figure S3). Other Fe deficiency response regulators (bHLH104, BRUTUS, PYE) exerted a less pronounced influence on coumarin profiles. Changes in these mutants were most obvious for sideritin glucoside and fraxin. Compared to WT, sideritin glucoside levels were increased in roots of *pye* and *bts* after P_i_ (-P_i_, +Fe) and combined deficiency (-P_i_, -Fe), respectively, whereas decreased levels were measured for *bhlh104* after combined deficiency. The most pronounced difference to WT was observed for fraxin, showing more than twofold higher levels in *bts* roots after Fe (+P_i_, -Fe) and combined deficiency (-P_i_, -Fe).

In addition to compare coumarin content between mutants and WT under each condition, differences in the responsiveness to the treatments between mutants and WT were evaluated. Fe deficiency response regulator mutants exhibited several statistically significant (two way ANOVA *P* ≤ 0.05) differences in the coumarin response to the treatments compared to WT plants (Supplementary Figure S4). In contrast to WT which responded to P_i_ deficiency in the presence of Fe (-P_i_, +Fe) with decreased levels of sideritin glucoside, *fit* plants reacted with a slight (1.8-fold) accumulation of sideritin glucoside levels (*P* = 0.0009 compared to +P_i_,+Fe conditions). P_i_ deficiency-induced esculin accumulation was absent in these plants. *Fit* plants were also impaired in the response to Fe deficiency with respect to sideritin glucoside and fraxin accumulation. This defect was more pronounced under P_i_ limiting conditions, where *fit* plants completely lost the ability to respond to Fe deficiency with sideritin glucoside accumulation, and even showed decreasing fraxin levels (-Fe/+Fe response at low P_i_). Furthermore, the reduction of Fe deficiency-induced sideritin glucoside levels by concomitant P_i_ limitation observed in WT plants, was absent in *fit* plants (-P_i_/+Pi response at -Fe). Under the same conditions, *fit* plants accumulated scopolin to a weaker extent than WT, and exhibited a decline in esculin levels. The P_i_ deficiency response in plants with impaired expression of *BRUTUS* affected the accumulation of scopoletin in the presence of Fe, whereas it was indistinguishable from WT in the absence of Fe. More differences in the response to Fe deficiency under P_i_ limiting condition could be observed. As such, *bts* plants exhibited a more pronounced accumulation of sideritin glucoside and fraxin, but no decline in esculin and scopoletin levels. Fe deficiency-induced fraxin accumulation was also enhanced under P_i_ sufficient conditions (16 *vs.* 7-fold, respectively, two way ANOVA *p* = 2 e^-5^). Plants deficient in the expression of *PYE* and *bHLH104* affected the response to Fe deficiency with respect to scopoletin accumulation at high P_i_ (*bhlh104*) and low P_i_ (*pye*) conditions. Additionally, Fe deficiency-induced sideritin accumulation was lower in *bhlh104* plants in the presence of low P_i_. Both mutants strongly responded to P_i_ deficiency with decreasing fraxin levels, but only in the presence of Fe. *Pye* mutants also failed to accumulate esculin under these conditions. The P_i_ deficiency response in the absence of Fe was indistinguishable from WT for both mutants.

### Effect of P_i_ Deficiency Response Regulator Mutants on Coumarin Profiles

**FIGURE 6:**
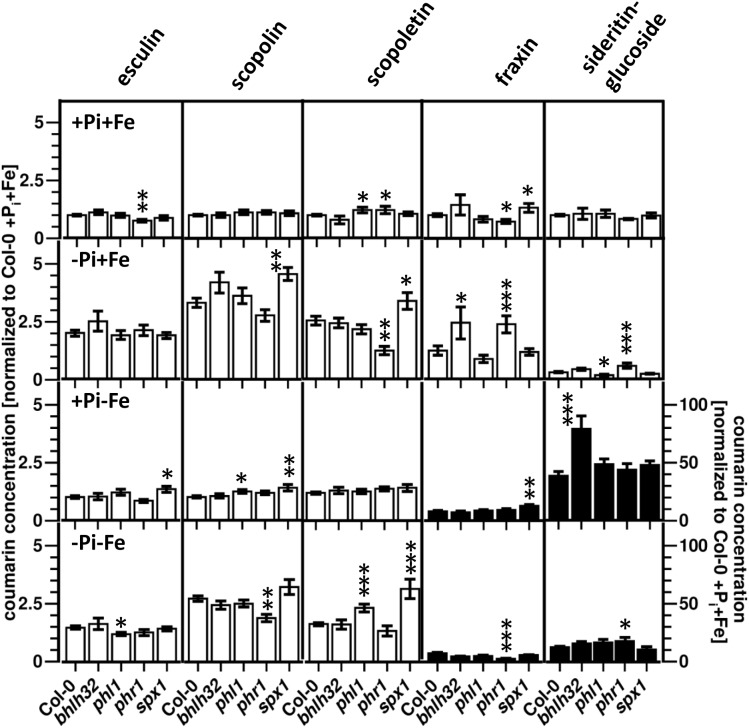
Normalized coumarin concentrations in roots of WT, *bhlh32*, *phl1*, *phr1*, and *spx1* plants (normalized to Col-0 +P_i_+Fe, average absolute amounts: esculin: 0.64 pmol mg^-1^ FW; scopolin: 702 pmol mg^-1^ FW; scopoletin: 16.8 pmol mg^-1^ FW; fraxin: 1.9 pmol mg^-1^ FW; sideritin glucoside: 3.6 pmol mg^-1^ FW). Plants were grown for 5 days on +P_i_+Fe agar plates, transferred to the indicated conditions and allowed to grow for additional 6 days before harvest. +P_i_: 500 μM, –P_i_: 5 μM, +Fe: 50 μM, –Fe: no Fe added. Error bars indicate SE (*n* ≥ 8). Significance analyses between mutants and Col0 were performed by Student’s *t*-test (two tailed, equal variances): ^∗^*P* ≤ 0.05; ^∗∗^*P* ≤ 0.01; ^∗∗∗^*P* ≤ 0.001. Note that the values for fraxin and sideritin glucoside in the lower two panels (black bars) refer to the y-axis to the right.

Of all four mutants with impaired expression of P_i_ deficiency response regulators, the *phr1* mutant exhibited the most comprehensive changes in coumarin profiles compared to WT (Figure 6 and Supplementary Figure S3). With respect to coumarin levels, *phr1* roots were most different to WT under P_i_ limiting conditions, in the presence as well as in the absence of Fe. In the presence of Fe, *phr1* plants exposed to P_i_ limitation (-P_i_, +Fe) exhibited higher levels of sideritin glucoside and fraxin, and lower levels of scopoletin compared to WT. In the additional absence of Fe (-P_i_, -Fe), sideritin glucoside levels were elevated, while scopolin and fraxin levels were lower. Fraxin as well as esculin concentrations in *phr1* roots were lower when plants were grown in P_i_ and Fe sufficient conditions (+P_i_, +Fe), whereas scopoletin content was increased. There were no statistically significant (*P* ≤ 0.05) changes in coumarin levels between *phr1* and WT roots in conditions of Fe deficiency in the presence of P_i_ (+P_i_, -Fe). The *spx1* mutant exhibited opposite effects compared to *phr1* with respect to the levels of fraxin under nutrient sufficient conditions (+P_i_, +Fe) as well as to the levels of scopoletin under P_i_ limiting conditions in the presence of Fe (-P_i_, +Fe). Additionally, aberrant *spx1* expression led to increased levels of esculin, scopolin, and fraxin in roots exposed to Fe deficiency (+P_i_, -Fe) and of scopoletin after exposure to combined deficiency (-P_i_, -Fe). In *bhlh32* plants, only the concentrations of the glucosides of the catechol type coumarins were altered, showing elevated levels of sideritin glucoside after Fe deficiency at high P_i_ (+P_i_, -Fe), and of fraxin after P_i_ deficiency in the presence of Fe (-P_i_, +Fe). Aberrant expression of the *phr1* homologue *phl1* only mildly affected coumarin profiles. These were most pronounced in roots exposed to combined Fe and P_i_ deficiencies (-P_i_, -Fe), and, interestingly, affected coumarins which were not affected in the *phr1* mutant, such as esculin and scopoletin.

Consistent with the changes in coumarin profiles, the coumarin response of *phr1* was also most different compared to WT for -P_i_ treatments, in the absence as well as in the presence of Fe (Supplementary Figure S4). As such, the P_i_ deficiency-induced decrease in sideritin glucoside levels observed in WT roots was less pronounced in *phr1* plants, but only in the presence of Fe. The effect of P_i_ deficiency in *phr1* plants on fraxin concentration depended on Fe availability. In the presence of Fe, P_i_ deficiency induced fraxin accumulation, whereas a reduction in fraxin levels was observed in the absence of Fe. Scopoletin levels, which were induced by P_i_ limitation in WT roots, were not affected in *phr1*. The Fe deficiency response of *phr1* in the presence of high Pi was indistinguishable from WT, but in P_i_ depleted conditions, the Fe deficiency-induced reduction in scopoletin levels was less pronounced. Also the response with respect to the accumulation of sideritin glucoside was lower (37-fold in WT vs. 29-fold in *phr1*). Compared to WT, low P_i_ treatment induced stronger scopolin and scopoletin accumulation in *spx1* in the presence and in the absence of Fe, respectively. However, differences in the -P_i_ response with respect to esculin and fraxin were only observed in the absence of Fe. The Fe deficiency response was also affected in *spx1*, but only in the presence of high P_i_. Here, induction of esculin levels was detected, which was not observed in WT, and fraxin accumulation was more pronounced. Interestingly, the majority of effects of plants with aberrant expression of the P_i_ deficiency response regulator *bHLH32* were detected after Fe deficiency. In the presence of high P_i_, Fe deficiency-induced sideritin glucoside accumulation was twice as strong in *bhlh32* plants compared to WT, whereas in low P_i_, a stronger reduction and weaker induction of scopolin and fraxin levels, respectively, was observed. Compared to WT, changes in the P_i_ deficiency response in *bhlh32* were only detectable in the absence of Fe, there only affecting sideritin glucoside levels. For *phl1*, different responses were observed for P_i_ deficiency-induced changes in the absence of Fe, exhibiting a lack of esculin accumulation and stronger induction of scopoletin levels, and for Fe deficiency-induced changes in low P_i_ conditions, showing unaltered scopoletin levels.

## Discussion

The impact of Fe-deficiency on root coumarin profiles has been well-established in recent years (Fourcroy et al., 2014, 2016; Schmid et al., 2014; Schmidt et al., 2014; Sisó-Terraza et al., 2016; Rajniak et al., 2018; Siwinska et al., 2018; Tsai et al., 2018). In order to cope with iron limiting conditions, Arabidopsis roots strongly accumulate coumarin glucosides, especially catechol-type coumarins glucosides, which are assumed to support Fe chelation and ferric ion reduction in order to facilitate Fe uptake after coumarin aglycones have been exuded to the rhizosphere. We recently showed an opposite effect of P_i_ deficiency on coumarin profiles, resulting in reduced exudation and reduced accumulation especially of catechol type coumarins and their glucosides (Ziegler et al., 2016). These observations prompted us to investigate coumarin profiles in Arabidopsis roots exposed to P_i_, Fe, and combined P_i_ and Fe deficiencies. Our results show that P_i_ deficiency resulted in lower sideritin glucoside and higher scopolin, scopoletin, and esculin levels. Furthermore, Fe deficiency-induced sideritin glucoside accumulation is dampened at low P_i_ conditions (-P_i_, -Fe), while scopolin, scopoletin, and esculin levels are slightly higher compared to Fe deficiency in the presence of high P_i_ (+P_i_, -Fe). These results indicate an antagonistic cross talk between Fe and P_i_ deficiency responses with respect to coumarin accumulation, which is most evident considering catechol-type coumarins. This cross-talk could be mediated by differences in the formation of Fe–P complexes, which depend on the concentrations of Fe and P_i_ supplied in the medium. As such, high P_i_ availability could lead to a relative decrease in available Fe, resulting in the accumulation of catechol-type coumarins. On the other hand, low P_i_ availability could lead to a relative increase in available Fe, which would result in decreased levels of catechol-type coumarins. These scenarios might apply to Fe sufficient conditions, in which relatively more iron would be available at low compared to high P_i_ concentrations in the medium, however, Fe availability in our Fe deficiency conditions with complete omission of any Fe sources should be independent of the external P_i_ concentration. Indeed, it was shown that P_i_ deficiency only led to increased iron concentrations in roots when plants were exposed to Fe sufficient conditions, whereas iron accumulation was not observed when plants were grown in the absence of Fe (Ward et al., 2008; Muller et al., 2015). Furthermore, if altered root Fe status because of changes in Fe–P complex formation would be the reason for P dependent changes in coumarin profiles, the levels catechol-type coumarins should be negatively correlated with P_i_ levels. However, compared to WT roots, sideritin glucoside and fraxin levels are lower in *pho1* roots exposed to nutrient sufficient and Fe deficient conditions, respectively, although P_i_ concentration is twice as high as in WT roots. Based on these results, we conclude that P dependent modifications of coumarin profiles are not solely due to altered Fe availability, and that P_i_ availability directly interferes with Fe deficiency-induced accumulation of catechol type coumarins.

The biosynthesis of coumarins proceeds via the generation of scopoletin/scopolin through the action of feruloyl CoA hydroxylase 1 (F6′H1), and subsequent hydroxylation reactions to fraxetin/fraxin and sideritin/sideritin glucoside (Kai et al., 2008; Rajniak et al., 2018; Siwinska et al., 2018; Tsai et al., 2018). Thus, P_i_ deficiency-induced accumulation of early coumarin intermediates, such as scopoletin/scopolin and esculin could be either due to increased biosynthesis, or to reduced conversion to coumarins exhibiting more elaborate hydroxylation patterns, or both. In most large scale gene expression studies, *F6′H1* mRNA levels were not altered in roots exposed to P_i_ deficiency (Bustos et al., 2010; Puga et al., 2014; Hoehenwarter et al., 2016; Mora-Macias et al., 2017). Instead, all studies reported strongly reduced expression of the gene At3G12900 (Bustos et al., 2010; Puga et al., 2014; Li and Lan, 2015; Hoehenwarter et al., 2016). This gene, whose expression and protein level are highly induced by Fe-deficiency, was recently characterized as scopoletin 8 hydroxylase (S8H), catalyzing the conversion of scopoletin to fraxetin (Rajniak et al., 2018; Siwinska et al., 2018; Tsai et al., 2018). We therefore assume that P_i_ deficiency-induced accumulation of scopolin and scopoletin is due to reduced conversion to downstream products. The increase in esculin might be a consequence of scopolin/scopoletin demethylation, although it is not clear yet, whether esculetin/esculin are actually derived from scopolin/scopoletin, or from caffeoyl CoA by a F6′H1-like reaction. Despite the reported P_i_ deficiency-induced reduction in *S8H* mRNA levels, our coumarin profiling did not show P_i_ deficiency-induced alterations in fraxin levels, neither in the presence nor in the absence of Fe. An explanation for this discrepancy could be that the subsequent hydroxylation step converting fraxetin/fraxin to sideritin/sideritin glucoside is impaired to a similar extent as the S8H reaction, which would lead to the observed constant fraxin level. Indeed, gene expression data generated in our group revealed a strong downregulation of the gene At4G31940 by P_i_ deficiency. At4G31940 encodes the P450 dependent monooxygenase CYP82C4 (Hoehenwarter et al., 2016). Reduced *CYP82C4* mRNA levels by P_i_ deficiency were also observed by other groups (Bustos et al., 2010; Li and Lan, 2015). Similar to *S8H* mRNA levels, *CYP82C4* mRNA levels strongly increase after Fe deficiency (Colangelo and Guerinot, 2004; Long et al., 2010; Lan et al., 2011; Rajniak et al., 2018). CYP82C4 protein was recently shown to catalyze the hydroxylation of fraxetin at position 5, yielding sideritin as product (Rajniak et al., 2018). Thus, the coumarin profiling data presented in this study and gene expression data from several studies suggest a P_i_ deficiency-induced negative regulation of the hydroxylation steps downstream of scopoletin/scopolin and fraxetin/fraxin, leading to reduced sideritin glucoside and increased scopoletin, scopolin, and esculin levels.

The analysis of mutants impaired in the expression of P_i_ and Fe deficiency response regulators revealed specificity of several transcription factors with respect to the nutrient conditions and to the biosynthesis of distinct coumarins, mainly of catechol type coumarins. The RING E3 ligase BRUTUS, known to negatively regulate Fe deficiency responses (Kobayashi et al., 2013; Selote et al., 2015; Zhang et al., 2015), also negatively regulates the accumulation of fraxin and sideritin glucoside, as suggested by the increased accumulation of both compounds in Fe depleted conditions in the *bts* mutant. Interestingly, Fe deficiency-induced hyper-accumulation of sideritin glucoside was only detectable in low P_i_ conditions. Thus, the reduction of Fe deficiency-induced sideritin glucoside levels by simultaneous P_i_ deficiency is less pronounced in *bts* plants compared to WT plants, indicating that the attenuation of Fe deficiency-induced sideritin glucoside accumulation by low P_i_ is mediated at least partially by BRUTUS. Whereas compared to WT, sideritin glucoside levels are elevated in roots of *bts*, they are reduced in *bhlh104* roots under conditions of combined P_i_ and Fe deficiency. This suggests that the proposed network consisting of the negative Fe deficiency response regulator BRUTUS, which targets bHLH104 (Selote et al., 2015), a positive regulator, is involved in the generation of coumarin profiles. However, the role of bHLH104 seems to be more complex, since it also affects fraxin levels under nutrient sufficient conditions. It was shown, that bHLH104 together with bHLH34 regulates the expression of PYE, as well as of bHLH38/39/100/101 transcription factors (Li et al., 2016), which contribute to FIT action by the formation of heterodimers (Colangelo and Guerinot, 2004; Yuan et al., 2008; Wang et al., 2013). The impact of the *fit* mutant is more pronounced compared to the *pye* mutant, suggesting that the FIT network represents the downstream target for BRUTUS and bHLH104 in the generation of coumarin profiles rather than PYE. FIT positively regulates mainly the accumulation of the catechol type coumarins sideritin glucoside and fraxin, which is corroborated by the absence of Fe deficiency-induced accumulation of *S8H* transcripts in *fit* plants (Colangelo and Guerinot, 2004). Interestingly, although FIT is known to regulate F6′H1 expression (Colangelo and Guerinot, 2004; Schmid et al., 2014), *fit* mutants still produce appreciable levels of scopolin, scopoletin, and esculin, indicating that other factors are involved in the control of constitutive levels of coumarins upstream of fraxin.

Of all tested P_i_ deficiency response regulators, bHLH32 and SPX1 seem to play a role in the generation of Fe deficiency-induced coumarin profiles, mainly in the presence of high P_i_. BHLH32 was identified as negative regulator of several P_i_ starvation responses (Chen et al., 2007). Our data showing increased sideritin glucoside levels in *bhlh32* mutants imply a role of this transcription factor also in the negative regulation of the biosynthesis of catechol type coumarins during Fe deficiency. SPX1, which interferes with the induction of P_i_ starvation responses (Puga et al., 2014; Wang et al., 2014), also regulates Fe deficiency-induced coumarin biosynthesis at high P_i_, as seen by the increased fraxin, esculin, and scopolin levels in *spx1* mutants. It is interesting that *phr1* plants do not show alteration of coumarin profiles in Fe depleted roots at high P_i_, since SPX1 was shown to exert its role by binding to PHR1 preventing its binding to the promoters of Pi starvation responsive genes (Puga et al., 2014; Wang et al., 2014). Possibly, SPX1 has additional targets, which are required to initialize the Fe deficiency-induced coumarin response at high P_i_ availability. However, the coumarin profiles suggest that P_i_ deficiency-induced changes in coumarin biosynthesis are mediated by PHR1, and to minor extent, by PHL1. Higher sideritin glucoside and fraxin levels in *phr1* plants grown on low P_i_/high Fe media indicate that downregulation of catechol type coumarin biosynthesis by P_i_ deficiency is controlled by PHR1. In low P_i_/Fe depleted media, *phr1* plants show increased sideritin glucoside, but decreased fraxin levels, which might be due to the loss of suppression of fraxin conversion suggesting that the conversion of fraxin to sideritin glucoside is more strongly controlled by PHR1 than the S8H reaction. It was already shown that modulation of expression of other Fe deficiency responsive genes by P_i_ starvation, such as *FERRIC REDUCTASE OXIDASE FRO3*, *IRON REGULATED TRANSPROTER IRT1*, IRT2, *NICOTIANAMINE SYNTHASE NAS1*, and *FRO6* was PHR1 and PHL1 dependent (Bustos et al., 2010). However, transcriptome data did not reveal PHR1 dependent downregulation of S8H and CYP82C4 by P_i_ deficiency (Bustos et al., 2010).

**FIGURE 7:**
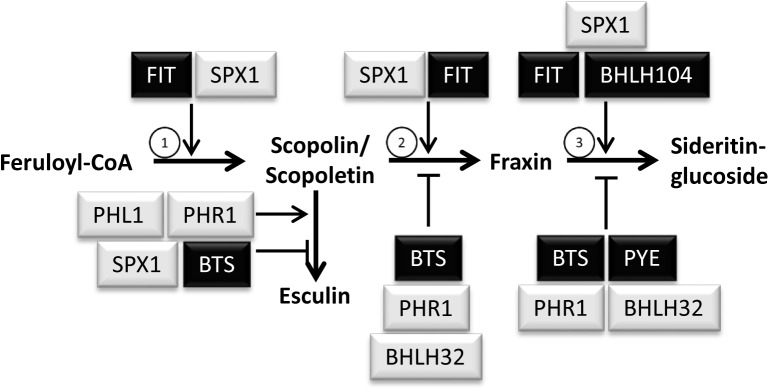
Tentative model depicting possible mode and site of action of P_i_ and Fe deficiency regulators in the regulation of coumarin profiles. Note that the model was drafted according to the coumarin profiling data generated in this study and is not supposed to indicate transcriptional control of individual biosynthetic steps. Regulators of Fe and P_i_ deficiency responses are shown in black and gray background, respectively. Encircled numbers indicate the enzymes catalyzing the respective step: (1) F6′H1; (2) S8H; (3) CYP82C4.

Taken together, several P_i_ and Fe deficiency response regulators are involved in the generation of Fe and P_i_ dependent coumarin profiles. A model was drafted summarizing possible modes and sites of action for each regulator irrespective of the growth condition (Figure 7). In this model, it was taken into account that changes in the level of coumarin compounds could be either due to changes in their biosynthesis, or to changes in their turnover, or to changes in the activity of the whole pathway.

According to this model, BRUTUS, PHR1, and bHLH32 negatively regulate both hydroxylation steps downstream of scopoletin/scopolin, whereas PYE seems to negatively impact solely the last hydroxylation reaction from fraxin to sideritin glucoside. The decreased levels of fraxin in *phr1* under nutrient sufficient conditions as well as under combined P_i_ and Fe deficiency was interpreted to be a consequence of higher fraxin to sideritin glucoside turnover. BHLH104, which is known to regulate several Fe starvation responses in an opposing way compared to BRUTUS, was placed in the model as a positive regulator of the conversion from fraxin to sideritin glucoside, based on decreased sideritin glucoside levels under conditions of combined P_i_ and Fe deficiency in *bhlh104* mutants. Increased fraxin levels observed in *bhlh104* in nutrient sufficient conditions, were interpreted as a consequence of impaired fraxin turnover to sideritin glucoside. FIT promotes both hydroxylation reactions leading to the generation of fraxin and sideritin glucoside and it also positively regulates the committed step in the pathway, the conversion of feruloyl CoA to scopoletin. Accumulation of scopoletin/scopolin in *phr1* and *phl1* also indicates positive regulation of this step by PHR1 and PHL1. However, since esculin concentrations are also reduced in both mutants, we rather assume that PHR1 and PHL1 might positively regulate the generation of esculin, which, if missing, indirectly might lead to the accumulation of scopoletin/scopolin. This is in contrast to the *bts* mutant, which exhibited increased esculin and decreased scopoletin levels, and which was therefore placed in the model as a negative regulator of esculin generation. The coumarin profiles in roots of *spx1* posed the major problem to fit this negative regulator of P_i_ deficiency responses into the model. Increased scopolin/scopoletin as well as esculin concentrations in *spx1* were interpreted as positive and negative regulation of scopolin/scopoletin and esculin generation, respectively, by SPX1, which would match the antagonistic interaction between PHR1 or PHL1 and SPX1. In contrast, if increased fraxin concentrations in *spx1* would be the consequence of impaired negative regulation of fraxin biosynthesis by SPX1, both SPX1 as well as PHR1 would be negative regulators of the same step. In order to preserve the antagonistic roles of both regulators, we decided to place SPX1 as a positive regulator of fraxin to sideritin glucoside conversion. However, we want to emphasize, that it is currently unknown whether the regulations shown in the model are due to transcriptional control of the respective genes. Only FIT was shown to regulate mRNAs levels for F6′H1, S8H, and CYP82C4 (Colangelo and Guerinot, 2004). As such, in the future, the elucidation of the molecular mechanism underlying the differential control of catechol-type coumarin biosynthesis will be interesting in order to see whether P_i_ and Fe deficiency response regulators such as PHR1 and FIT independently inactivate or activate the transcription of respective genes. The presence of the P_i_ deficiency responsive *PHR1 Binding site* (*P1BS*) and the Fe deficiency responsive IDRS *cis*-acting element in the AtFER1 promoter suggests that transcriptional control of this gene by Fe as well as by P_i_ deficiency is mediated by binding of Fe and P_i_ deficiency response regulators to different sites of the promoter (Bournier et al., 2013). It remains to be elucidated whether this also applies to the differential regulation of coumarin biosynthesis genes, or, alternatively, whether P_i_ and Fe deficiency response regulators might interact on the protein level, thus interfering with each other in the activation of the promoters.

## Availability of Data and Material

Metabolite profiling data have been deposited to the EMBL-EBI MetaboLights database (doi: 10.1093/nar/gks1004. PubMed PMID: 23109552) with the identifier MTBLS831.

The complete dataset can be accessed here https://www.ebi.ac.uk/metabolights/MTBLS831.

## Author Contributions

RC designed the study, performed most of the measurements and biological experiments, and co-wrote the manuscript. JZ designed the study, supported the measurements, and wrote the manuscript. SA designed the study and co-wrote the manuscript. All authors have read and approved the final version of the manuscript.

## Conflict of Interest Statement

The authors declare that the research was conducted in the absence of any commercial or financial relationships that could be construed as a potential conflict of interest.

## Supplementary Material

Figure S1 Chemical structures of coumarins measured in this study. ^a^Note that the position of the glucoside (R5, R7, or R8) in sideritin glucoside has not been confirmed yet by NMR studies.

Figure S2 (A) Pictures of WT and mutant seedlings 6 days after transfer from +P_i_+Fe (5 days) to +P_i_+Fe. The height of each panel corresponds to 7 cm. +P_i_: 500 μM; +Fe: 50 μM. **(B)** Pictures of WT and mutant seedlings 6 days after transfer from +P_i_+Fe (5 days) to -P_i_+Fe. The height of each panel corresponds to 7 cm. +P_i_: 500 μM; +Fe: 50 μM; -P_i_: 5 μM. **(C)** Pictures of WT and mutant seedlings 6 days after transfer from +P_i_+Fe (5 days) to +P_i_-Fe. The height of each panel corresponds to 7 cm. +P_i_: 500 μM; +Fe: 50 μM; -Fe: no Fe added. **(D)** Pictures of WT and mutant seedlings 6 days after transfer from +P_i_+Fe (5 days) to -P_i_-Fe. The height of each panel corresponds to 7 cm. +P_i_: 500 μM; +Fe: 50 μM; -P_i_: 5 μM; -Fe: no Fe added.

Figure S3 Coumarin concentrations in roots of mutants relative to Col-0 (Col-0 set to one). The fold changes compared to Col-0 are color coded according to the scale bar. Gray squares indicate no difference to Col0-0 at *P* ≤ 0.05 (Student’s *t*-test, paired, two sided).

Figure S4 Coumarin response **(A)** upon P_i_ starvation in the presence or absence of Fe, and **(B)** upon Fe deficiency in the presence of high (500 μM) or low (5 μM) P_i_. The fold changes compared to the respective nutrient sufficient conditions are color coded. For Col-0, only changes at *P* ≤ 0.05 (Student’s *t*-test, two tailed equal variance) are color coded. For the mutants, the fold changes compared to the respective nutrient sufficient conditions are color coded, if the response was different compared to the WT response at *P* ≤ 0.05 (two way ANOVA). Gray indicates no differences.

TABLE S1 Primers used for genotyping.

TABLE S2 MS parameters for MRM transitions.

DATASET S1 Raw data of all the analysis presented in this study.
