# Supplementary material for: Iron and Phosphate Deficiency Regulators Concertedly Control Coumarin Profiles in Arabidopsis thaliana Roots During Iron, Phosphate, and Combined Deficiencies
Source: Front Plant Sci. 2019 Feb 11;10:113. doi: 10.3389/fpls.2019.00113 (PMC6378295; doi:10.3389/fpls.2019.00113)
Supplement: Figure S1 — Chemical structures of coumarins measured in this study. aNote that the position of the glucoside (R5, R7, or R8) in sideritin glucoside has not been confirmed yet by NMR studies. [file Data_Sheet_1.PDF]

Supplemental Figure 1

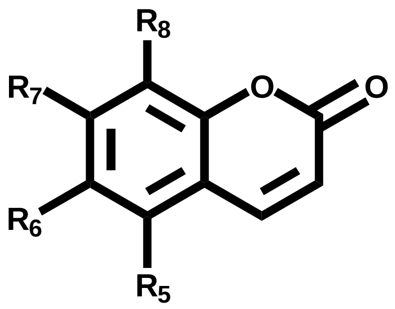

name	R_5	R_6	R_7	R_8
Sideritin-Glucoside[a]	OH	OCH_3	O-Glucose	OH
Esculin	H	O-Glucose	OH	H
Esculetin	H	OH	OH	H
Fraxin	H	OCH_3	OH	O-Glucose
Scopolin	H	OCH_3	O-Glucose	H
Scopoletin	H	OCH_3	OH	H

Supplemental Figure 1: Chemical structures of coumarins measured in this study. [a] Note that the position of the glucoside (R_5, R_7, or R_8) in sideritin glucoside has not been confirmed yet by NMR studies.
