# Supplementary material for: Iron and Phosphate Deficiency Regulators Concertedly Control Coumarin Profiles in Arabidopsis thaliana Roots During Iron, Phosphate, and Combined Deficiencies
Source: Front Plant Sci. 2019 Feb 11;10:113. doi: 10.3389/fpls.2019.00113 (PMC6378295; doi:10.3389/fpls.2019.00113)
Supplement: Figure S2 — (A) Pictures of WT and mutant seedlings 6 days after transfer from +Pi+Fe (5 days) to +Pi+Fe. The height of each panel corresponds to 7 cm. +Pi: 500 μM; +Fe: 50 μM. (B) Pictures of WT and mutant seedlings 6 days after transfer from +Pi+Fe (5 days) to -Pi+Fe. The height of each panel corresponds to 7 cm. +Pi: 500 μM; +Fe: 50 μM; -Pi: 5 μM. (C) Pictures of WT and mutant seedlings 6 days after transfer from +Pi+Fe (5 days) to +Pi-Fe. The height of each panel corresponds to 7 cm. +Pi: 500 μM; +Fe: 50 μM; -Fe: no Fe added. (D) Pictures of WT and mutant seedlings 6 days after transfer from +Pi+Fe (5 days) to -Pi-Fe. The height of each panel corresponds to 7 cm. +Pi: 500 μM; +Fe: 50 μM; -Pi: 5 μM; -Fe: no Fe added. [file Data_Sheet_2.PDF]

Supplemental Figure 2

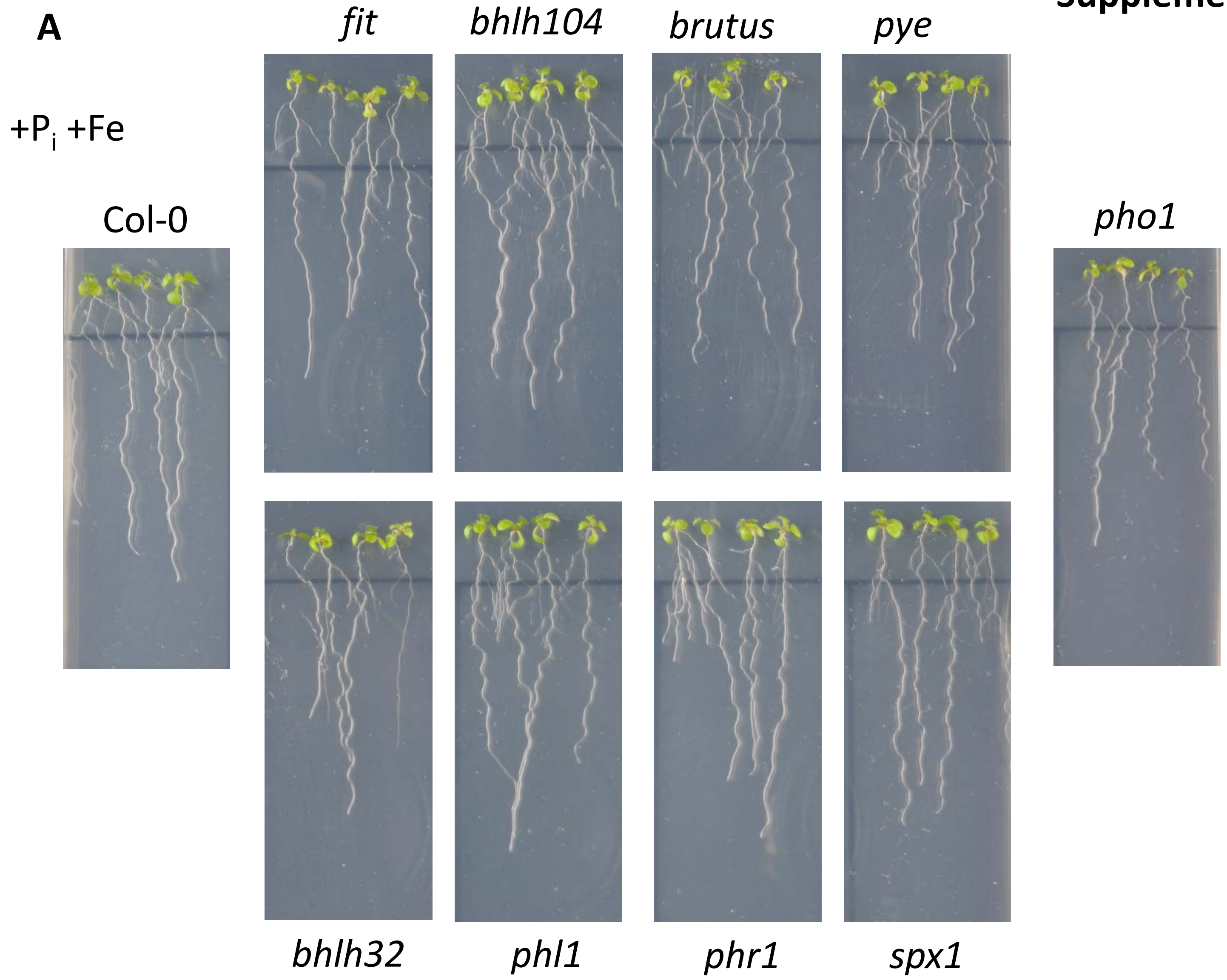

Supplemental Figure 2A: Pictures of WT and mutant seedlings 6 days after transfer from +P_i+Fe (5 days) to +P_i+Fe. The height of each panel corresponds to 7 cm. +P_i: 500 µM; +Fe: 50 µM

Supplemental Figure 2

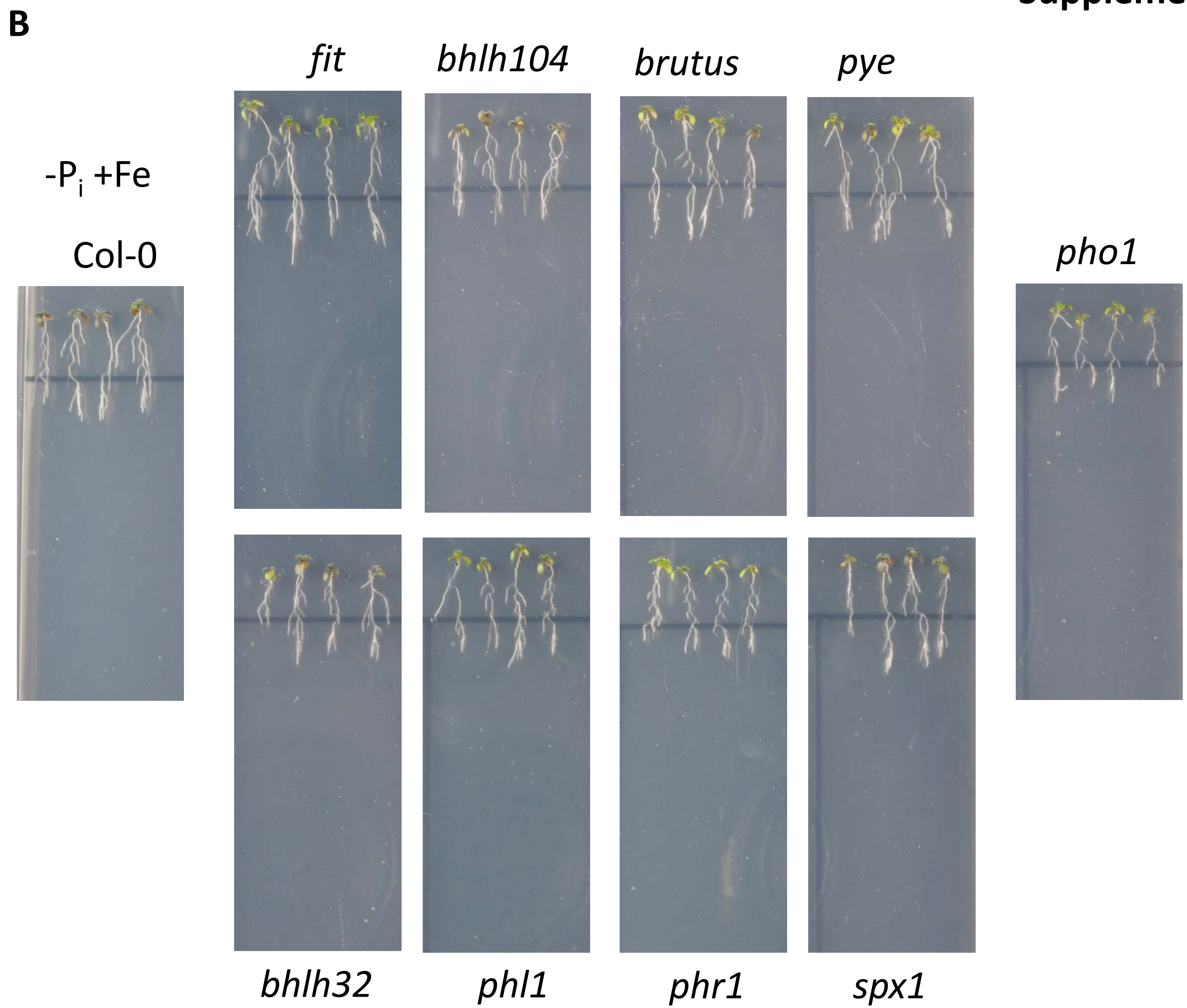

Supplemental Figure 2B: Pictures of WT and mutant seedlings 6 days after transfer from $+P_i+Fe$ (5 days) to $-P_i+Fe$. The height of each panel corresponds to 7 cm. $+P_i$: 500 µM; +Fe: 50 µM; $-P_i$: 5 µM

Supplemental Figure 2

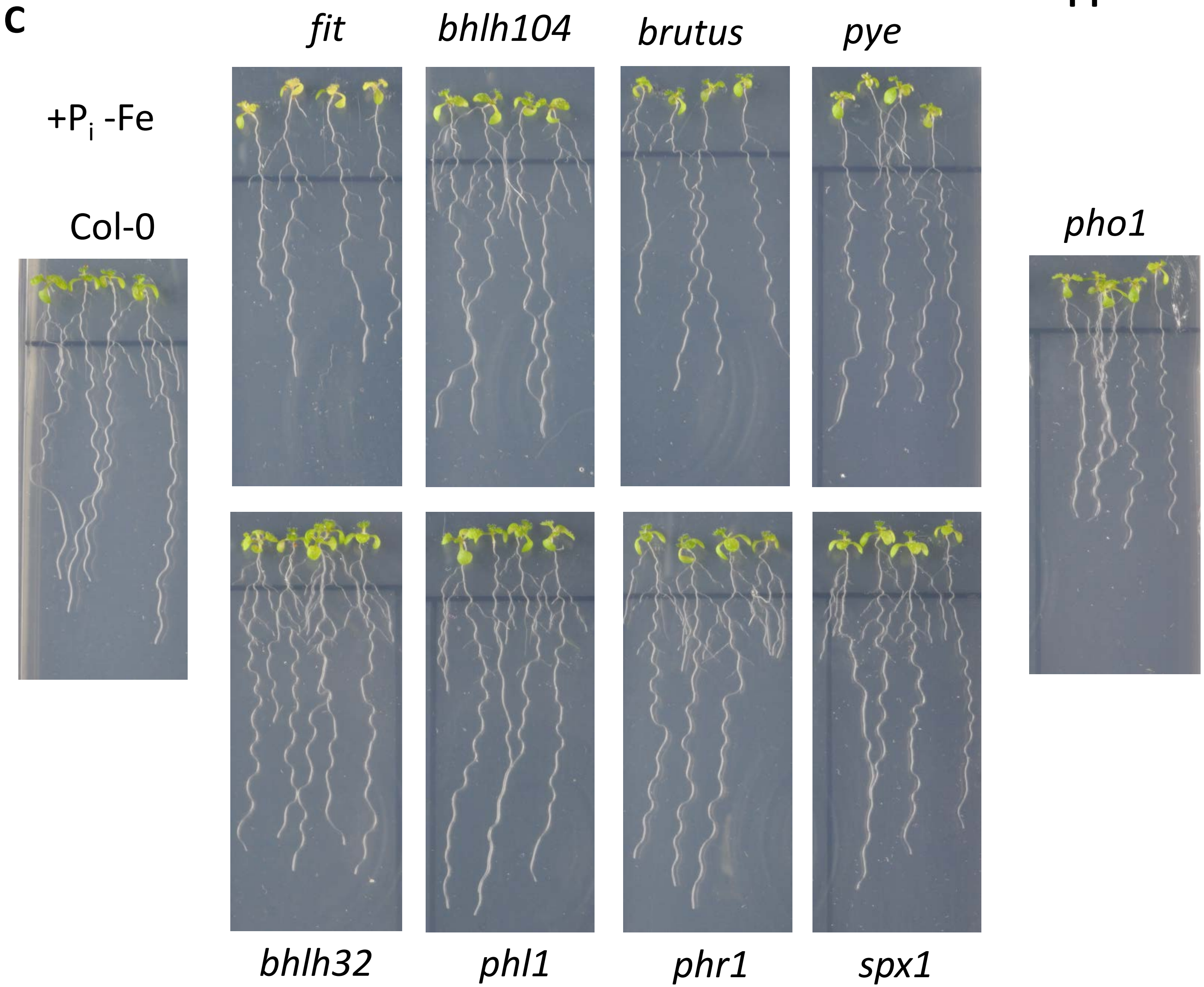

Supplemental Figure 2C: Pictures of WT and mutant seedlings 6 days after transfer from $+P_i$+Fe (5 days) to $+P_i$-Fe. The height of each panel corresponds to 7 cm. $+P_i$: 500 µM; +Fe: 50 µM; -Fe: no Fe added

Supplemental Figure 2

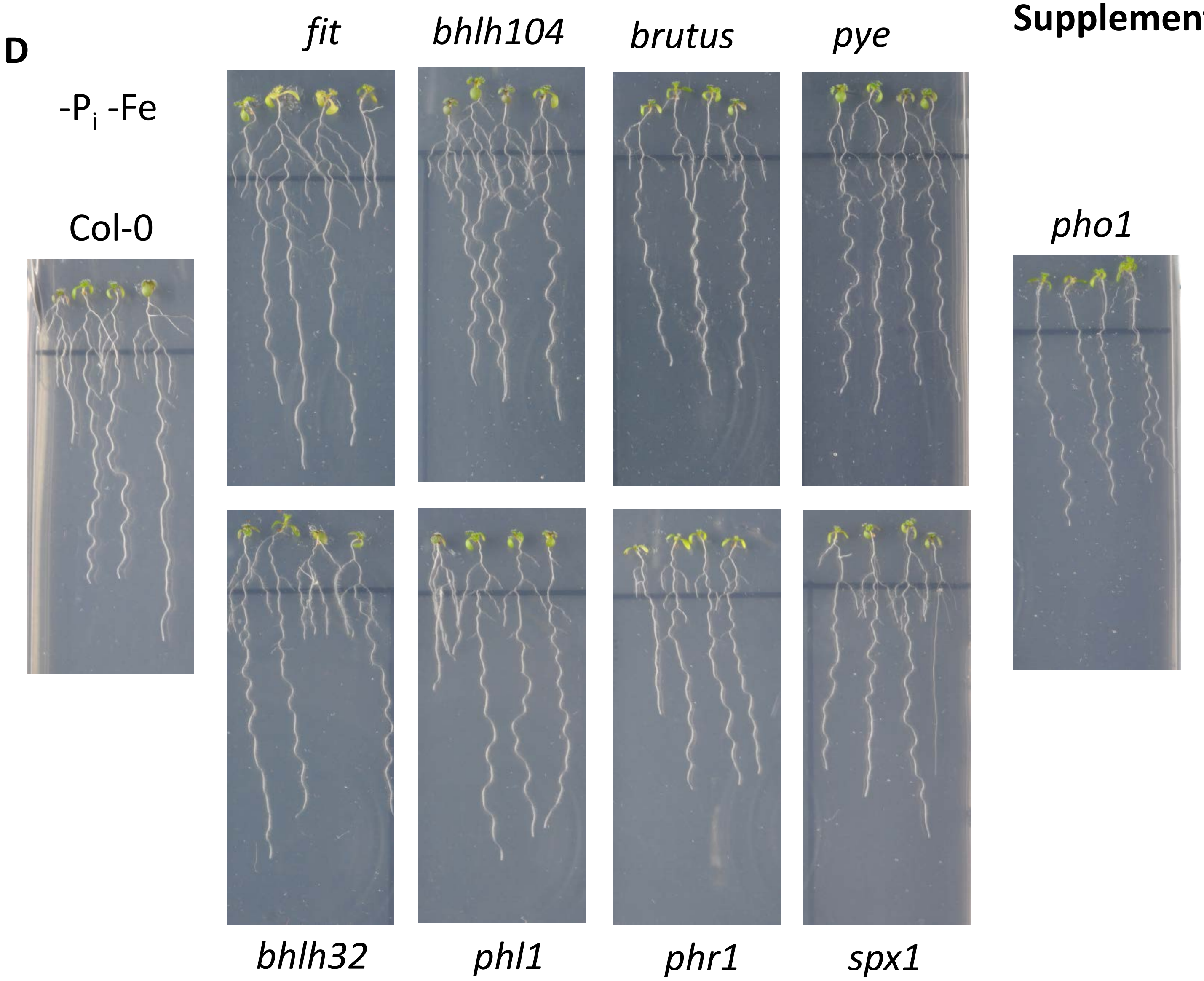

Supplemental Figure 2D: Pictures of WT and mutant seedlings 6 days after transfer from $+P_i$+Fe (5 days) to $-P_i$-Fe. The height of each panel corresponds to 7 cm. $+P_i$: 500 µM; +Fe: 50 µM; $-P_i$: 5 µM; -Fe: no Fe added
