# Supplementary material for: Iron and Phosphate Deficiency Regulators Concertedly Control Coumarin Profiles in Arabidopsis thaliana Roots During Iron, Phosphate, and Combined Deficiencies
Source: Front Plant Sci. 2019 Feb 11;10:113. doi: 10.3389/fpls.2019.00113 (PMC6378295; doi:10.3389/fpls.2019.00113)
Supplement: Figure S3 — Coumarin concentrations in roots of mutants relative to Col-0 (Col-0 set to one). The fold changes compared to Col-0 are color coded according to the scale bar. Gray squares indicate no difference to Col0-0 at P ≤ 0.05 (Student’s t-test, paired, two sided). [file Data_Sheet_3.PDF]

Supplemental Figure 3

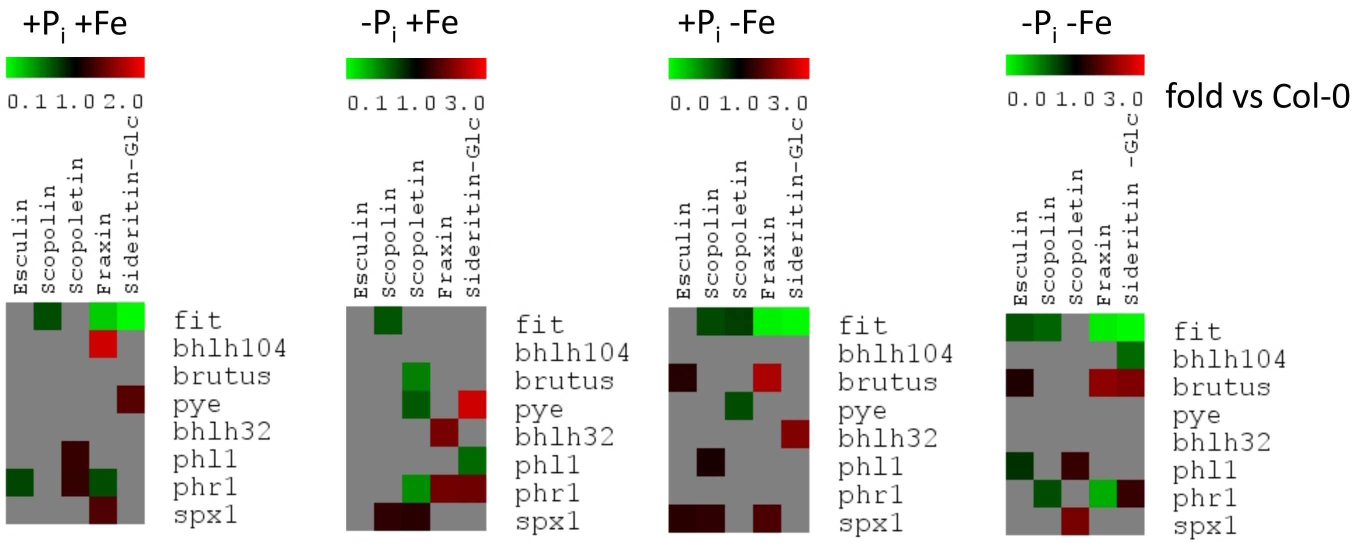

Supplemental Figure 3: Coumarin concentrations in roots of mutants relative to Col-0 (Col-0 set to one) . The fold changes compared to Col-0 are color coded according to the scale bar. Gray squares indicate no difference to Col0-0 at $P \leq 0.05$ (Student's *t*-test, paired, two sided)
