# Supplementary material for: Iron and Phosphate Deficiency Regulators Concertedly Control Coumarin Profiles in Arabidopsis thaliana Roots During Iron, Phosphate, and Combined Deficiencies
Source: Front Plant Sci. 2019 Feb 11;10:113. doi: 10.3389/fpls.2019.00113 (PMC6378295; doi:10.3389/fpls.2019.00113)
Supplement: Figure S4 — Coumarin response (A) upon Pi starvation in the presence or absence of Fe, and (B) upon Fe deficiency in the presence of high (500 μM) or low (5 μM) Pi. The fold changes compared to the respective nutrient sufficient conditions are color coded. For Col-0, only changes at P ≤ 0.05 (Student’s t-test, two tailed equal variance) are color coded. For the mutants, the fold changes compared to the respective nutrient sufficient conditions are color coded, if the response was different compared to the WT response at P ≤ 0.05 (two way ANOVA). Gray indicates no differences. [file Data_Sheet_4.PDF]

Supplemental Figure 4

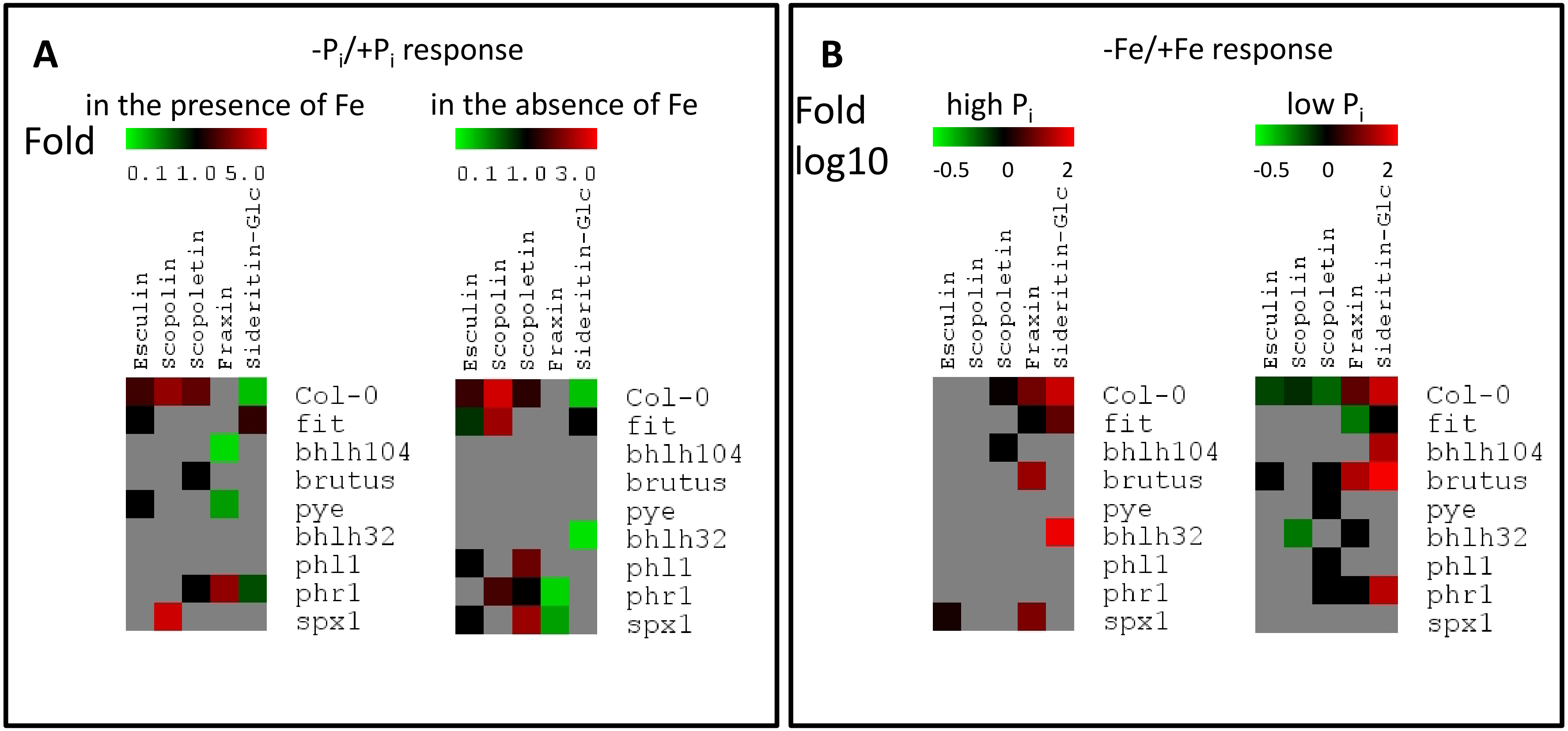

Supplemental Figure 4: Coumarin response A: upon P_i starvation in the presence or absence of Fe, and B: upon Fe deficiency in the presence of high (500 µM) or low (5 µM) P_i.
The fold changes compared to the respective nutrient sufficient conditions are color coded.
For Col-0, only changes at $P \leq 0.05$ (Student's *t*-test, two tailed equal variance) are color coded.
For the mutants, the fold changes compared to the respective nutrient sufficient conditions are color coded, if the response was different compared to the WT response at $P \leq 0.05$ (two way ANOVA). Grey indicates no differences.
