# Supplementary material for: Iron and Phosphate Deficiency Regulators Concertedly Control Coumarin Profiles in Arabidopsis thaliana Roots During Iron, Phosphate, and Combined Deficiencies
Source: Front Plant Sci. 2019 Feb 11;10:113. doi: 10.3389/fpls.2019.00113 (PMC6378295; doi:10.3389/fpls.2019.00113)
Supplement: TABLE S1 — Primers used for genotyping. [file Data_Sheet_5.PDF]

Supplemental Table 1: Primers used for genotyping

Genotype	Gene identifier	T-DNA insertion line	mutant allele described in	Primer	
bhlh32	**AT3G25710**	SALK_013517	Chen et al., 2007	LP-bhlh32	5'-TTTGCCTCTTAATACCCCCTG-3'
				RP-bhlh32	5'-TGAGTGCACAAGAAGTCATGG-3'
phr1-3	**AT4G28610**	SALK_067629	Nilsson et al., 2007	LP-PHR1	5'-GAGAGACCTCACACGCACTTC-3'
				RP-PHR1	5'-CTTTCTGGCGAACCTGTAGTG-3'
phl1-2	**AT5G29000**	SALK_079505	Bournier et al., 2013	LP-PHL1	5'-GTGGAGACGTTTCTGCACTTC-3'
				RP-PHL1	5'-GGAGTAAGGGTGGACTCATCC-3'
spx1	**AT5G20150**	SALK_039445	Duan et al., 2008	LP-SPX1	5'-AACCTCTTCCCCCTCTCTTTC-3'
				RP-SPX1	5'-TGCTCCAACAATGGAATCTTC-3'
pye-1	**AT3G47640**	SALK_021217	Long et al. 2010	LP-PYE1	5'-GTGTCTGGGGATCAGGTTGT -3'
				RP-PYE1	5'-ATCGTCTGATGAAGCAAATGC-3'
bts-1	**AT3G18290**	SALK_016526	Long et al. 2010	LP-BTS	5'-CCAAATGCGTTCGTAGGTAAG-3'
				RP-BTS	5'-TCAGATTTACACAAATTTGCAGC-3'
fit-3 (fruG108)	**AT2G28160**	GK-108C10	Jakoby et al., 2004	LP-fit	5'-TTGACGAAAACCCAGTTCTTG-3'
				RP-fit-GK	5'-AGCATGTTCTTGGTACCCTCC-3'
pho1	**AT3G23430**	SAIL_423_E04	this study	LP-PHO1	5'-ATCCTCGTGTTCTTCCACATG-3'
				RP-PHO1	5'-CAACGAGGAGCAAAACAAAAG-3'
bhlh104	**AT4G14410**	SALK_099496	Zhang etal. 2015	LP-BHLH104	5'-GGGGAAAGGTTGTGTCTTTTG-3'
				RP-BHLH104	5-'GCCTGAGTTCTTGATCACGAG-3'

T-DNA border Primer:	LBb1.3	5'-ATTTTGCCGATTTCGGAAC-3'
	LB1-SAIL	5'-GCCTTTTCAGAAATGGATAAATAGCCTTGCTTCC-3'
	LB-GK-o8409	5'-ATATTGACCATCATACTCATTGC-3'

Bournier M, Tissot N, Mari S, Boucherez J, Lacombe E, Briat JF, Gaymard F: **Arabidopsis ferritin 1 (AtFer1) gene regulation by the phosphate starvation response 1 (AtPHR1) transcription factor reveals a direct molecular link between iron and phosphate homeostasis**. *The Journal of biological chemistry* 2013, **288**(31):22670-22680.

Chen ZH, Nimmo GA, Jenkins GI, Nimmo HG: **BHLH32 modulates several biochemical and morphological processes that respond to Pi starvation in Arabidopsis**. *The Biochemical journal* 2007, **405**(1):191-198.

Duan K, Yi K, Dang L, Huang H, Wu W, Wu P: **Characterization of a sub-family of Arabidopsis genes with the SPX domain reveals their diverse functions in plant tolerance to phosphorus starvation**. *The Plant journal : for cell and molecular biology* 2008, **54**(6):965-975.

Jakoby M, Wang HY, Reidt W, Weisshaar B, Bauer P: **FRU (BHLH029) is required for induction of iron mobilization genes in Arabidopsis thaliana**. *FEBS letters* 2004, **577**(3):528-534.

Long TA, Tsukagoshi H, Busch W, Lahner B, Salt DE, Benfey PN: **The bHLH transcription factor POPEYE regulates response to iron deficiency in Arabidopsis roots**. *The Plant cell* 2010, **22**(7):2219-2236.

Nilsson L, Muller R, Nielsen TH: **Increased expression of the MYB-related transcription factor, PHR1, leads to enhanced phosphate uptake in Arabidopsis thaliana**. *Plant, cell & environment* 2007, **30**(12):1499-1512.

Zhang J, Liu B, Li M, Feng D, Jin H, Wang P, Liu J, Xiong F, Wang J, Wang HB: **The bHLH transcription factor bHLH104 interacts with IAA-LEUCINE RESISTANT3 and modulates iron homeostasis in Arabidopsis**. *The Plant cell* 2015, **27**(3):787-805.
