# Supplementary material for: Iron and Phosphate Deficiency Regulators Concertedly Control Coumarin Profiles in Arabidopsis thaliana Roots During Iron, Phosphate, and Combined Deficiencies
Source: Front Plant Sci. 2019 Feb 11;10:113. doi: 10.3389/fpls.2019.00113 (PMC6378295; doi:10.3389/fpls.2019.00113)
Supplement: TABLE S2 — MS parameters for MRM transitions. [file Data_Sheet_6.PDF]

Supplemental Table 2 MS parameters for MRM-transitions

	MRM transitions	Retention time, min	ionization energy, V	collision energy, V
succinate-D4	**267→177**	2.97	-135	10
	267→251			10
phosphate	**315→299**	2.84		20
	315→225			30

Quantifier and qualifier transitions are indicated in bold and italics, respectively
